# Transjugular intrahepatic portosystemic shunt for the treatment of portal hypertensive biliopathy with cavernous transformation of the portal vein: a case report

**DOI:** 10.1186/s12876-022-02168-2

**Published:** 2022-03-03

**Authors:** Ming Zhao, Xiaoze Wang, Bangxi Liu, Xuefeng Luo

**Affiliations:** Department of Gastroenterology and Hepatology, Sichuan University-University of Oxford Huaxi Joint Centre for Gastrointestinal Cancer, West China Hospital, Sichuan University, Chengdu, Sichuan China

**Keywords:** Portal hypertensive biliopathy, Transjugular intrahepatic portosystemic shunt, Cavernous transformation of the portal vein

## Abstract

**Background:**

Portal hypertensive biliopathy (PHB) was caused by anatomical and functional abnormalities in the intrahepatic and extrahepatic bile ducts secondary to portal hypertension. Currently, there is no consensus regarding to the optimal treatment for PHB. Transjugular intrahepatic portosystemic shunt (TIPS) is the treatment choice for the management of symptomatic PHB, however, it could be very difficult in patients with PHB and cavernous transformation of portal vein.

**Case presentation:**

We report a case of PHB, successfully managed with TIPS. A 23-year-old man with liver cirrhosis presented with jaundice. Magnetic resonance cholangiopancreatography (MRCP) showed multiple tortuous hepatopetal collateral vessels compressing the common bile duct (CBD) and leading to the dilated proximal bile duct. He was diagnosed with PHB and treated with TIPS. A guidewire was inserted into the appropriate collateral vessel through transsplenic approach to guide intrahepatic puncture and TIPS was performed successfully. After the operation, portal vein pressure decreased and the symptoms of biliary obstruction were relieved significantly. In addition, the patient showed no jaundice at a follow-up of one year.

**Conclusions:**

For PHB patients presenting for cavernous transformation of the portal vein, which precludes the technical feasibility of TIPS, a combined transjugular/transsplenic approach could be an alternative option.

## Background

Generally, portal hypertensive biliopathy (PHB) was caused by abnormalities in the intra- or extrahepatic bile ducts secondary to portal hypertension and commonly accompanied with extrahepatic portal venous obstruction (EHPVO). Only 5–30% of patients show symptoms of fever, abdominal pain, jaundice, and skin itching [1–4]. There is no consensus regarding to the optimal treatment for PHB. Endoscopic, surgical and interventional treatment are the treatment options for patients with symptomatic PHB [5–7]. Individualized treatment maybe decided case-by-case.

Some studies have reported that transjugular intrahepatic portosystemic shunt (TIPS) could be used for symptomatic PHB, but it is very challenging in patients with cavernous transformation of portal vein [8–11]. In this report, we present a case of EHPVO-related PHB that was alleviated by TIPS via a combined transjugular/transsplenic approach.

## Case presentation

**Fig. 1:**
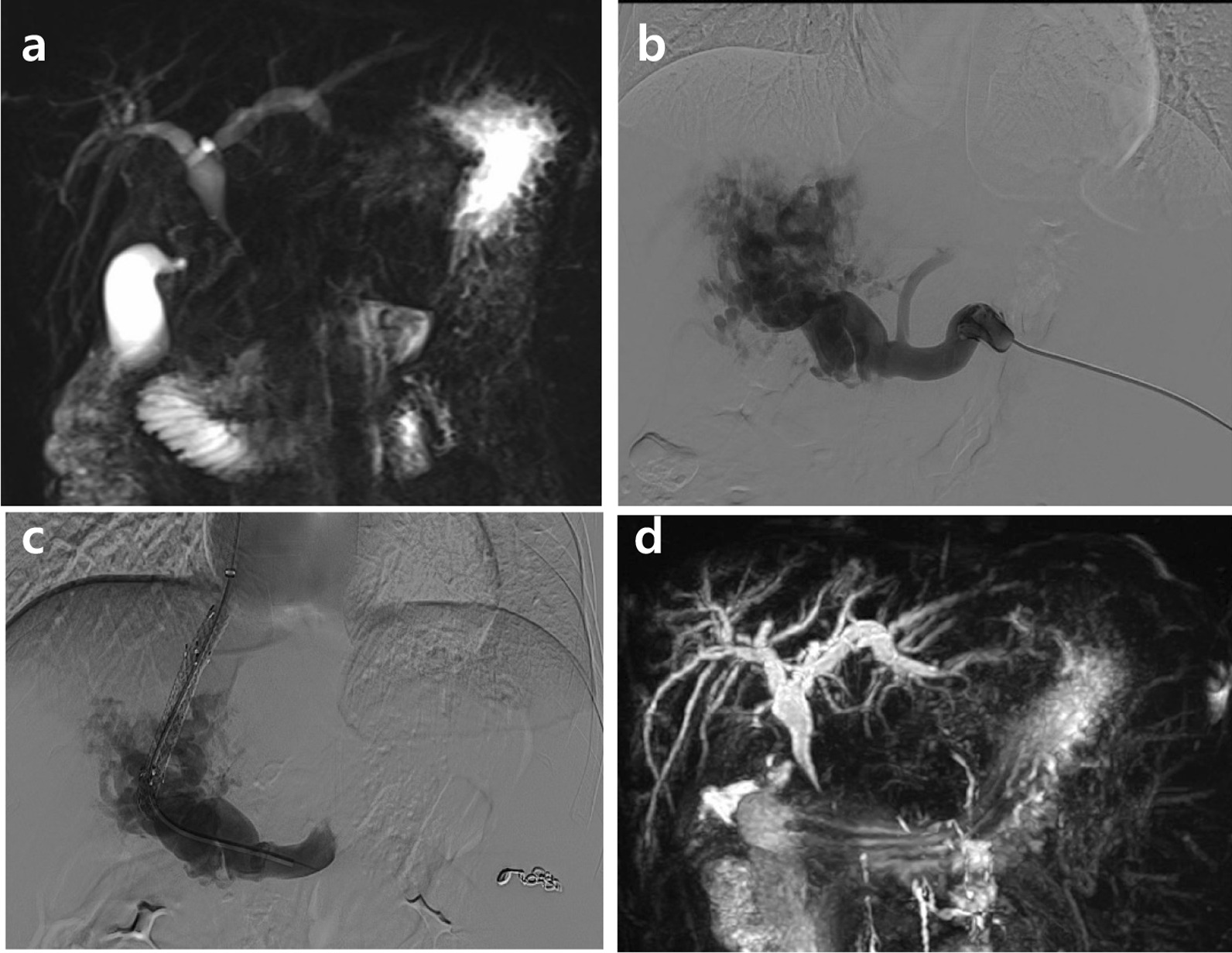
MRCP images before and after TIPS. **a** MRCP images showed multiple tortuous hepatopetal collateral vessels compressing the common bile duct, and leading to the dilated proximal bile duct before operation. **b** Portography performed before TIPS via a transsplenic access demonstrated cavernomatous transformation of the portal vein. **c** Portography performed after TIPS showed good outflow in the stent. **d** The biliary obstruction was alleviated after TIPS

A 23-year-old man with liver cirrhosis presented with jaundice for one month. The initial laboratory test indicated total bilirubin (TB) of 4.81 mg/dL, direct bilirubin (DB) of 4.44 mg/dL, alanine aminotransferase of 268 U/L, aspartate aminotransferase of 128 U/L, alkaline phosphatase(ALP) of 1105 U/L, and gamma-glutamyl transferase (GGT) of 1018 U/L. Child–Pugh’s classification was graded as A. Gastroscopy showed moderate esophageal and gastric varices. Magnetic resonance cholangiopancreatography (MRCP) showed multiple tortuous hepatopetal collateral vessels compressing the common bile duct (CBD) and leading to the dilated proximal bile duct (Fig. 1a). TIPS was considered as alternative approach to decrease portal venous pressure and alleviate bile obstruction. The main trunk and intrahepatic branches of portal vein were completely occluded and replaced by collaterals. Most collaterals were small, torturous and not suitable for TIPS placement. Traditional TIPS based on cross-sectional images may not guarantee that the suitable collateral was punctured. Therefore, a guidewire was inserted into the appropriate collateral vessel through transsplenic approach to guide intrahepatic puncture to ensure a linear intrahepatic shunt (Fig. 1b). Once the collateral vein was accessed successfully, indirect portography was performed. The intrahepatic tract was dilated using a balloon catheter to allow the implantation of an 8 mm × 60 mm expanded polytetrafluoroethylene covered stent. The portosystemic pressure gradient decreased from 24 to 13 mmHg and an 6 mm coil was used to embolize the splenic access after withdrawal of the sheath (Fig. 1c). Three months after the operation, TB dropped to 2.23 mg/dL, DB to 1.39 mg/dL, ALP to 504 U/L and GGT to 670 U/L. A follow-up MRCP revealed that the biliary obstruction was alleviated (Fig. 1d). In addition, the patient showed no jaundice.

## Discussion and conclusions

Although the pathogenesis of PHB is not fully elucidated, it has been postulated that external pressure caused by dilated porto-porto collaterals and/or ischaemic strictures of the bile duct may the may reason. In the present case, biliary obstruction was caused by compression of the collaterals due to EHPVO. Surgical shunt, which was gradually replaced by TIPS, has proven to feasible and effective in patients with EHPVO and complications of portal hypertension. However, it is very difficult since the intrahepatic portal branch was totally occluded. During TIPS procedure, it is vital to identify the appropriate collateral to insure the good outflow in the stent. Transsplenic access and retrograde catheterization would make it easier. Habib et al. [12] demonstrated the feasibility of transsplenic TIPS in 11 patients with chronic portal vein thrombosis with a success rate of 100%. We also embolized the transsplenic tract to avoid the risk of perisplenic hemorrhage.

Endoscopic treatment is preferred in patients with CBD stones, cholangitis or patients with dominant biliary stricture, but without a shuntable vein. It includes endoscopic sphincterotomy, stone extraction, and biliary stricture dilatation with or without stent or nasobiliary drain placement [6, 9, 13]. In this case, MRCP showed dominant biliary stricture without CBD stones and cholangitis. It may be risky if endoscopic treatment such as biliary stricture dilatation with or without stent in the presence of collaterals in the region. The patient presented with jaundice and moderate esophageal and gastric varices and the shuntable vein was present. Therefore, TIPS was considered as alternative approach to decrease portal venous pressure and alleviate bile obstruction and performed successfully. After the operation, portal vein pressure decreased and the symptoms of biliary obstruction were relieved significantly. Therefore, for PHB patients with cirrhosis, presenting for cavernous transformation of the portal vein, which precludes the application of TIPS, the combined transjugular/transsplenic approach can be used as an alternative treatment.

## Data Availability

Data sharing is not applicable to this article as no datasets were generated or analysed during the current study.

## Abbreviations

PHB: Portal hypertensive biliopathy
TIPS: Transjugular intrahepatic portosystemic shunt
EHPVO: Extrahepatic portal venous obstruction
TB: Total bilirubin
DB: Direct bilirubin
ALP: Alkaline phosphatase
GGT: Gamma-glutamyl transferase
MRCP: Magnetic resonance cholangiopancreatography
CBD: Common bile duct

## References

[1] Dilawari JB, Chawla YK (1992). Pseudosclerosing cholangitis in extrahepatic portal venous obstruction. Gut.

[2] Khuroo MS, Yattoo GN, Zargar SA (1993). Biliary abnormalities associated with extrahepatic portal venous obstruction. Hepatology.

[3] Condat B, Vilgrain V, Asselah T (2003). Portal cavernoma-associated cholangiopathy: a clinical and MR cholangiography coupled with MR portography imaging study. Hepatology.

[4] Sezgin O, Oğuz D, Altintaş E (2003). Endoscopic management of biliary obstruction caused by cavernous transformation of the portal vein. Gastrointest Endosc.

[5] Wallner BK, Schumacher KA, Weidenmaier W (1991). Dilated biliary tract: evaluation with mr cholangiography with a t2-weighted contrast-enhanced fast sequence. Radiology.

[6] Chattopadhyay S, Nundy S (2012). Portal biliopathy. World J Gastroenterol.

[7] Suarez V, Puerta A, Santos LF (2013). Portal hypertensive biliopathy: a single center experience and literature review. World J Hepatol.

[8] Bayraktar Y, Öztürk MA, Egesel T (2000). Disappearance of “pseudocholangiocarcinoma sign” in a patient with portal hypertension due to complete thrombosis of left portal vein and main portal vein web after web dilatation and transjugular intrahepatic portosystemic shunt. J Clin Gastroenterol.

[9] Oo YH, Olliff S, Haydon G (2009). Symptomatic portal biliopathy: a single centre experience from the UK. Eur J Gastroenterol Hepatol.

[10] Cellich PP, Crawford M, Kaffes AJ (2015). Portal biliopathy: multidisciplinary management and outcomes of treatment. ANZ J Surg.

[11] Gorgul A, Kayhan B, Dogan I, et al. Disappearance of the pseudocholangiocarcinoma sign after TIPSS. Am. J. Gastroenterol 1996; 150–154 8561119

[12] Habib A, Desai K, Hickey R (2015). Portal vein recanalization-transjugularintrahepatic portosystemic shunt using the transsplenic approach to achieve transplant candidacy in patients with chronic portal vein thrombosis. J Vasc Interv Radiol.

[13] Dhiman RK, Behera A, Chawla YK (2007). Portal hypertensive biliopathy. Gut.

